# Rack1 mediates tyrosine phosphorylation of Anxa2 by Src and promotes invasion and metastasis in drug-resistant breast cancer cells

**DOI:** 10.1186/s13058-019-1147-7

**Published:** 2019-05-22

**Authors:** Yanling Fan, Weiyao Si, Wei Ji, Zhiyong Wang, Zicong Gao, Ran Tian, Weijie Song, He Zhang, Ruifang Niu, Fei Zhang

**Affiliations:** 1 Public Laboratory, Tianjin Medical University Cancer Institute and Hospital, National Clinical Research Center for Cancer, Tianjin, 300060 China; 2 Key Laboratory of Cancer Prevention and Therapy, Tianjin, 300060 China; 3 Tianjin’s Clinical Research Center for Cancer, Tianjin, 300060 China; 4 Key Laboratory of Breast Cancer Prevention and Therapy, Ministry of Education, Tianjin, 300060 China

**Keywords:** Rack1, Anxa2, Src, Drug resistance, Invasion, Metastasis, Breast cancer

## Abstract

**Background:**

Acquirement of resistance is always associated with a highly aggressive phenotype of tumor cells. Recent studies have revealed that Annexin A2 (Anxa2) is a key protein that links drug resistance and cancer metastasis. A high level of Anxa2 in cancer tissues is correlated to a highly aggressive phenotype. Increased Anxa2 expression appears to be specific in many drug-resistant cancer cells. The functional activity of Anxa2 is regulated by tyrosine phosphorylation at the Tyr23 site. Nevertheless, the accurate molecular mechanisms underlying the regulation of Anxa2 tyrosine phosphorylation and whether phosphorylation is necessary for the enhanced invasive phenotype of drug-resistant cells remain unknown.

**Methods:**

Small interfering RNAs, small molecule inhibitors, overexpression, loss of function or gain of function, rescue experiments, Western blot, wound healing assays, transwell assays, and in vivo metastasis mice models were used to investigate the functional effects of Rack1 and Src on the tyrosine phosphorylation of Anxa2 and the invasion and metastatic potential of drug-resistant breast cancer cells. The interaction among Rack1, Src, and Anxa2 in drug-resistant cells was verified by co-immunoprecipitation assay.

**Results:**

We demonstrated that Anxa2 Tyr23 phosphorylation is necessary for multidrug-resistant breast cancer invasion and metastasis. Rack1 is required for the invasive and metastatic potential of drug-resistant breast cancer cells through modulating Anxa2 phosphorylation. We provided evidence that Rack1 acts as a signal hub and mediates the interaction between Src and Anxa2, thereby facilitating Anxa2 phosphorylation by Src kinase.

**Conclusions:**

Our findings suggest a convergence point role of Rack1/Src/Anxa2 complex in the crosstalk between drug resistance and cancer aggressiveness. The interaction between Anxa2 and Rack1/Src is responsible for the association between drug resistance and invasive/metastatic potential in breast cancer cells. Thus, our findings provide novel insights on the mechanism underlying the functional linkage between drug resistance and cancer aggressiveness.

**Electronic supplementary material:**

The online version of this article (10.1186/s13058-019-1147-7) contains supplementary material, which is available to authorized users.

## Background

Resistance is associated with or promotes a highly aggressive phenotype [1]. One common mechanism is that cancer cells can evolve additional capabilities, such as enhanced invasion and metastasis with the acquisition of resistance. These abilities are often associated with reprogramming of intracellular gene expression and activation of the corresponding intracellular signaling pathways [2–5]. For instance, metastasis-related proteins are often upregulated in drug-resistant cells [1, 2, 6–8]; chronic anticancer drug treatments often induce epithelial-to-mesenchymal transition in tumor cells, a phenotypic change that is closely related to metastasis [9–11]. Therefore, the drug resistance of cancer cells not only leads to treatment failure, but may also result in rapid recurrence and metastasis. Thus, determining the molecular mechanisms governing the aggressive behavior of drug-resistant tumors is critical for designing highly effective therapeutic strategies.

Recent studies have revealed several key molecules involved in the development of drug resistance and associated signal pathways responsible for cancer progression [2, 12]. One molecule is Annexin A2 (Anxa2), a calcium-dependent phospholipid-binding protein [2, 12–14]. Anxa2 has been shown as a multifunctional protein implicated in many biological processes [13–15]. Its abnormal expression is associated with a variety of diseases, especially cancer [13–15]. Anxa2 overexpression promotes proliferation, migration, invasion, angiogenesis, and metastasis in various types of tumors [13–15]. In addition, a high Anxa2 level has been observed in many types of drug-resistant cells [16–20]. The increased expression of Anxa2 in these cells not only confers resistance to anticancer agents, but also enhances their aggressive behavior [18, 21, 22]. Moreover, clinical studies have shown that Anxa2 overexpression is positively correlated to poor response to anticancer agents and rapid recurrence in cancer patients who had received chemotherapy [21, 23–25]. This evidence suggests that Anxa2 is a key protein that links drug resistance and cancer metastasis. Therefore, uncovering the detailed mechanisms through which Anxa2 promotes cancer progression is urgently required.

The biological function of Anxa2 is modulated by post-translational modifications, including phosphorylation, acetylation, ubiquitination, and sumoylation [13, 15, 26]. Anxa2 can be phosphorylated at Tyr23 by Src-family tyrosine kinase in response to growth factors, such as epidermal growth factor (EGF) and platelet-derived growth factor (PDGF) [13]. In addition, Anxa2 Tyr23 phosphorylation (pY23-Anxa2) has been found to be upregulated in response to anticancer drugs, including genotoxic agents and microtubule interfering agents [12, 27]. pY23-Anxa2 is involved in the promotion of invasion and metastasis in cancer cells and associated with disease progression in cancer patients [13, 15]. Nevertheless, little information is available concerning the regulatory mechanism of Anxa2 tyrosine phosphorylation, and whether the phosphorylation is necessary for the enhanced invasive phenotype of drug-resistant cells remains unknown.

We have previously demonstrated that the receptor for activated protein C kinase 1 (Rack1) is a novel binding protein of Anxa2 [28]. Rack1 acts as a multifaceted scaffold protein that is involved in various cellular activities by mediating protein–protein interactions [29, 30]. The function of Rack1 on cancer progression is cancer type-specific [29–31]. Except for colon and gastric cancer [32–34], Rack1 appears to play a tumor-promoting role in other carcinomas [29–31]. Additionally, Rack1 overexpression has been correlated to proliferation, invasion, and metastasis [35–38]. In particular, higher Rack1 level is an independent predictor for poor prognosis in breast cancer. Recently, Rack1 has been reported to be a key protein involved in drug resistance in several carcinomas [39–43]. These findings have suggested that Rack1 may act as an important convergence point for drug resistance and invasion/metastasis. We previously reported that Rack1 is required for the migration and invasion potential of drug-resistant breast cancer cells [28]. We demonstrated that Rack1 acts as a molecular bridge mediating the binding of Src and Anxa2 to P-glycoprotein. Interestingly, Rack1 is also a binding protein of Src kinase [30]. Rack1 knockdown decreases Adriamycin-triggered Tyr23 phosphorylation of Anxa2 in drug-resistant breast cancer [28]. However, the detailed mechanism by which Rack1 regulates Anxa2 phosphorylation remains unclear. The accurate function of Rack1 in promoting the aggressive behavior in drug-resistant breast cancer cells has not been thoroughly determined. In this work, we further explored the effect of Rack1 on the invasive and metastatic potential in drug-resistant breast cancer cells, and investigated the mechanism underlying the regulation of Anxa2 Tyr23 phosphorylation. We showed that Rack1 and Anxa2 are required for the invasive and metastatic potential of multidrug-resistant (MDR) breast cancer cells. Rack1 mediates Anxa2 binding to Src, thereby facilitating Anxa2 phosphorylation by Src. Our findings suggest that the interaction between Anxa2 and Rack1/Src is responsible for the association between drug resistance and aggressive behavior in breast cancer cells.

## Materials and methods

### Antibodies, reagents, and drugs

The following antibodies, reagents, and drugs were used: DMEM/F12, RPMI 1640, DMEM/High glucose medium and trypsin (Hyclone, Logan, UT, USA); Fetal Bovine Serum (FBS, Gibco, Carlsbad, CA, USA); Src inhibitor KX2-391 (Selleckchem, Houston, TX, USA); transwell inserts (Corning Inc., Corning, NY, USA); EGF (Cat No. 236-EG, R&D, USA); Matrigel (BD Biosciences, San Jose, CA, USA); Protein A/G agarose beads (Invitrogen, Carlsbad, CA, USA); Lipofectamine RNAiMax and Lipofectamine 2000 (Invitrogen, Carlsbad, CA, USA); antibodies against Rack1 (sc-17754), Anxa2 (sc-28385), p-Anxa2 (sc-135753), GFP (sc-9996, Santa Cruz Biotechnology, Santa Cruz, CA, USA); antibodies against Src (# 2123) and p-Src (# 6943, Cell Signaling Technology, Beverly, MA, USA); antibodies against Flag (F1804) and β-actin (A1978, Sigma, St. Louis, MO, USA); and Rack1, Anxa2, and Src small interfering RNAs (siRNAs, Invitrogen, Carlsbad, CA, USA, detailed information is listed in Table 1).

**Table 1:** siRNA sequences used in this study

Name	Sequence
siRack1-1#	Upper: UAUCUCGAGAUCCAGAGACAAUCUG
	Lower: CAGAUUGUCUCUGGAUCUCGAGAUA
siRack1-2#	Upper: ACGAUGAUAGGGUUGCUGCUGUUGG
	Lower: CCAACAGCAGCAACCCUAUCAUCGU
siSrc-1#	Upper: CAGCAGCUGGUGGCCUACUACUCCA
	Lower: UGGAGUAGUAGGCCACCAGCUGCUG
siSrc-2#	Upper: GAGCCCAAGCUGUUCGGAGGCUUCA
	Lower: UGAAGCCUCCGAACAGCUUGGGCUC
siAnxa2-1#	Upper: UACAGCAGCGCUUUCUGGUAGUCGC
	Lower: GCGACUACCAGAAAGCGCUGCUGUA

### Cell culture and siRNA transfection

Human triple-negative breast cancer (TNBC) MDA-MB-468 cells were obtained from American Type Culture Collection (ATCC). The drug-resistant variant cell line MDA-MB-468/EPR was established by our group using a stepwise exposure to an increasing concentration of epirubicin. The human luminal-type breast cancer MCF-7 cells and its drug-resistant cell line MCF-7/ADR was kindly provided by Dr. Zizheng Hou (Henry Ford Hospital, Detroit, MI, USA). Anxa2 stably silenced MCF-7/ADR and control cells have been established in our previous study [18]. HEK-293T cells were obtained from ATCC. The following media supplemented with 10% FBS were used for cell culture: DMEM/F12 for MDA-MB-468/EPR, modified RPMI-1640 for MCF-7/ADR, and DMEM/High glucose for HEK-293T. For siRNA transfection, cells were seeded into six-well plates and cultured to 30–40% confluence. Afterwards, control, Rack1-, Anxa2-, and Src-specific siRNAs were transfected using Lipofectamine RNAiMAX reagent according the manufacturer’s instructions.

### Vector construction, lentivirus production, and infection

Flag-tagged, full-length, and wild-type Rack1 (Rack1^WT^) was generated by polymerase chain reaction (PCR) amplification with the following primers: upper: 5′-GAGAGCTAGCATGACTGAGCAGATGACCCT-3′, lower: 5′- GAGAGCGGCCGCCTACTTGTCGTCATCGTCTTTGTAGTCGCGTGTGCCAATGG-3′. Afterwards, the Rack1-coding region was cloned into a lentiviral vector pCDH-hygromycin in the Nhe I and Not I cloning sites. The Rack1^Y246F^ mutant was generated by mutating the codon TAC (Y) of its amino acid 246 to TTC (F), where tyrosine 246 was replaced by phenylalanine. For Rack1 stable knockdown, a validated siRNA sequence targeting the Rack1 non-coding region (5′-TGGCACACGCTAGAAGTTTATGG-3′) was cloned into the lentiviral vector pLKO.1-hygromycin of the BamH I and Age I cloning sites. The lentiviral plasmids pCDH-Anxa2^WT^-GFP, pCDH-Anxa2^Y23A^-GFP, and pCDH-Anxa2^Y23D^-GFP were obtained in our previous study. In brief, wild-type Anxa2 (Anxa2^WT^) was generated by PCR amplification with the following primers: upper: 5′-CGGCTCGAGATGTCTACTGTTCACGAAATCCTG-3′, lower: 5′-CGTGGATCCGTCATCTCCACCACACAG-3′. Afterwards, the Anxa2-coding region was cloned into pEGFP-N3 vector in the Xho I and BamH I cloning sites. Anxa2^Y23A^-GFP and Anxa2^Y23D^-GFP mutants were generated by mutating the codon TAT (Y) of its amino acid 23 to GCT (A) or GAT (D). Then, the Anxa2-GFP fragments were further subcloned into the lentiviral vector pCDH-hygromycin in the Nhe I and Not I cloning sites. All constructs were confirmed by double-enzyme digestion and DNA sequencing. Lentivirus production was performed as described previously by using a three-plasmid packaging system. In brief, HEK-293T cells were plated in 10-cm dish and cultured to 60% confluence. Afterwards, the cells were co-transfected with the lentiviral plasmid and two packaging plasmids. The virus in the supernatants were collected, concentrated, and used to infect target cells 48 h after transfection. Stable cell lines were selected by using 50 μg/mL of hygromycin B.

### Western blot and co-immunoprecipitation assay

Western blot assay was performed as described previously [44]. Briefly, the cells were lysed with 1× SDS lysis buffer; the cell lysates after protein quantification were used for SDS-PAGE separation and subsequently transferred to a PVDF membrane. The membranes were blocked by 5% non-fat milk and probed with corresponding primary antibodies. After washing with 1× TBST, the membrane was incubated with horseradish peroxidase-labeled secondary antibodies, and the signals were subsequently detected using the ECL kit. β-actin was used as a loading control. For EGF-induced Anxa2 phosphorylation assay, control, Rack1, or Src siRNA transfected cells were starved for 8 h. Then, the cells were stimulated with 10 ng/mL of EGF for 0, 5, and 10 min. The cells were lysed, and the total cellular protein was analyzed by using Western blotting analysis. Co-IP assay was performed as described previously [28]. Briefly, MCF-7/ADR and MDA-MB-468/EPR cells were transfected with negative control, Src-, Anxa2-, or Rack1-specific siRNAs for 72 h. Then, the cells were lysed with Tris-Trion X100-based lysis buffer. Afterwards, the quantified cell lysates were pre-cleared with protein G-linked agarose beads, followed by incubation with corresponding antibodies (Anxa2, Rack1, or Src) to enrich immunocomplex. The interacting proteins were captured with Protein A/G conjugated beads and subsequently analyzed by Western blotting with anti-Anxa2, Rack1, or Src antibodies.

### Wound healing and transwell assay

Wound healing assay was carried out as described previously [12]. The control and experimental cells were planted in six-well plates and grown to confluence. Afterward, a wound was prepared by scraping the cell monolayer using a 10-μL pipette tip. Afterward, the cells were incubated at 37 °C in a medium containing 0.5% FBS, and the cells were allowed to migrate for 0, 12, 24, 36, 48, and 60 h. The wound areas were photographed under an inverted microscope in indicated times, and the relative migrated distance was calculated with ImageJ. Transwell assays were performed by using Boyden chambers with a 8-μm filter pore. For migration assay, 1 × 10^5^ cells in 200 μL serum-free medium were loaded into the upper chamber. The lower chamber was loaded with 600 μL of 10% FBS-containing medium. After incubation at 37 °C for 16 h, the migrated cells were fixed, stained, and quantified at 200×. For invasion assay, 2.5 × 10^5^ cells were loaded into the upper insert coated with Matrigel, and the incubation time was 24 h. To examine the effect of Src inhibitors on migration and invasion of drug-resistant cells, the cells were pre-treated with Src inhibitors for 2 h. Then, transwell assays were performed in the absence (for migration) or presence (for invasion) of Matrigel.

### In vivo metastasis assay

Six-week-old female SCID mice were purchased from Beijing Charles River. All operations followed the guidelines approved by the Animal Ethical and Welfare Committee of Tianjin Medical University Cancer Institute and Hospital. For metastasis assay, control and Rack1 stably silenced cells, as well as the Rack1 rescued cells, were cultured to log phase. Then, the cells were trypsinized, washed thrice with PBS, resuspended, and adjusted to a concentration of 2 × 10^7^ cells/mL. 2 × 10^6^ cells (*n* = 8 per group) were injected into SCID mice via tail veins. Three months after injection, the mice were sacrificed; the lungs were dissected, fixed with formalin, embedded in paraffin, and serially sectioned. After hematoxylin and eosin staining, the micrometastasis foci in lungs were visualized and counted under a microscope.

### Immunofluorescence staining and apoptosis assay

Standard protocols are detailed in Additional file 1: Supplementary methods.

### Statistical analysis

All data were shown as mean ± SD. The statistical differences were analyzed using the GraphPad Prism 7.00 software (GraphPad Software, La Jolla, CA, USA). For multiple sets of comparisons, one-way or two-way ANOVA was performed. *P* values less than 0.01 (two-tailed) were considered statistically significant. *indicates that the *P* value is less than 0.01 and ** means *P* value is less than 0.001.

## Results

### Rack1 is required for Anxa2 Tyr23 phosphorylation and enhanced invasiveness of drug-resistant breast cancer cells

**Fig. 1:**
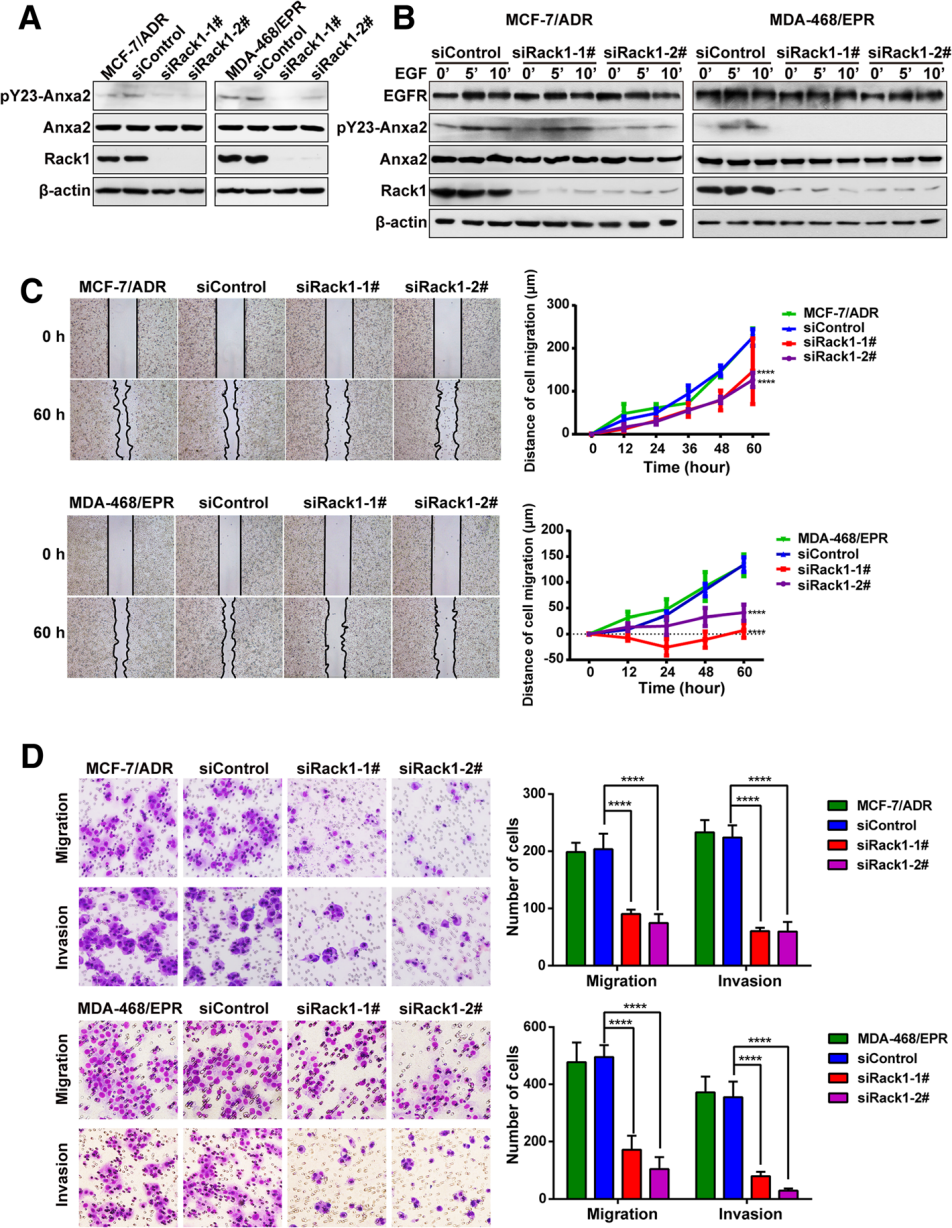
Rack1 is required for Anxa2 Tyr23 phosphorylation and enhanced invasiveness of drug-resistant breast cancer cells. **a** Rack1 knockdown decreased the basal levels of phosphorylated Anxa2 in two drug-resistant cells. Western blotting analysis of the total and phosphorylated Anxa2 expression in MCF-7/ADR and MDA-MB-468/ERP cancer cells transfected with negative control or siRNAs targeting Rack1 for 72 h; β-actin was used as the loading control. **b** Rack1 knockdown inhibited EGF-induced Tyr23 phosphorylation of Anxa2. **c** Knockdown of Rack1 expression in two drug-resistant cells significantly decreased cell migration ability as measured by wound healing assay. Data are shown as mean ± SD; *n* = 6; ****P < 0.0001 versus control. Statistical analysis was performed by two-way ANOVA. **d** Knockdown of Rack1 expression attenuated the migration and invasion ability in two drug-resistant cells. For cell migration assay, 1 × 10^5^ cells in 200 μL of serum-free medium were loaded into the upper chamber. For cell invasion assay, 2.5 × 10^5^ cells in 200 μL serum-free medium were loaded into the upper chamber coated with Matrigel. The statistical results are summarized in the right panel. Data as mean ± SD; *n* = 6; ****P < 0.0001 compared with the control group

To determine whether Rack1 is necessary for Anxa2 tyrosine phosphorylation, we silenced the expression of Rack1 in two drug-resistant breast cancer cell lines using two different Rack1-specific siRNAs. As shown in Fig. 1a, Rack1 expression was remarkably downregulated in Rack1 siRNA transfected cells compared with that of the control siRNA transfected group. The level of pY23-Anxa2 was notably decreased in Rack1-silenced cells than in the control cells. Anxa2 tyrosine phosphorylation can be induced by growth factors, such as EGF [13, 15]. We examined the effect of Rack1 knockdown on EGF-induced Anxa2 phosphorylation. As shown in Fig. 1b, Rack1 knockdown attenuated the increase of pY23-Anxa2 induced by EGF in two drug-resistant cells, while the effect of Rack1 silencing on pY23-Anxa2 was evident in MDA-MB-468/EPR cells compared to MCF-7/ADR cells. This variance may be due to the differences in the genetic background between the two cell lines, such as the expression level of endogenous EGFR (Additional file 2: Figure S1), which is higher in MCF-7/ADR cells. Next, we further investigated the function linkage between Rack1 knockdown and cell migration and invasion ability. As shown in Fig. 1c, the knockdown of Rack1 expression in two drug-resistant cells significantly decreased cell migration ability as measured by wound healing assay. Similarly, the results from transwell assay showed that the migration and invasion abilities were significantly inhibited in Rack1-silenced cells compared with control cells (Fig. 1d). To exclude the effect of cell death on migration and invasion, we investigated the effect of Rack1 knockdown on the apoptosis of resistant cells by flow cytometry using Annexin V-FITC/PI double staining method. As shown in Additional file 2: Figure S2, silencing the expression of Rack1 had no significant effect on apoptosis in resistant cells compared to control cells. Therefore, the decrease of cell migration/invasion ability after Rack1 knockdown is not due to the increased incidence of cell death. Collectively, these data demonstrated that Rack1 silencing inhibited Anxa2 tyrosine phosphorylation along with decreased cell migration and invasion abilities.

### Inhibition of Src kinase blocked Anxa2 tyrosine phosphorylation and decreased invasiveness of MDR breast cancer cells

**Fig. 2:**
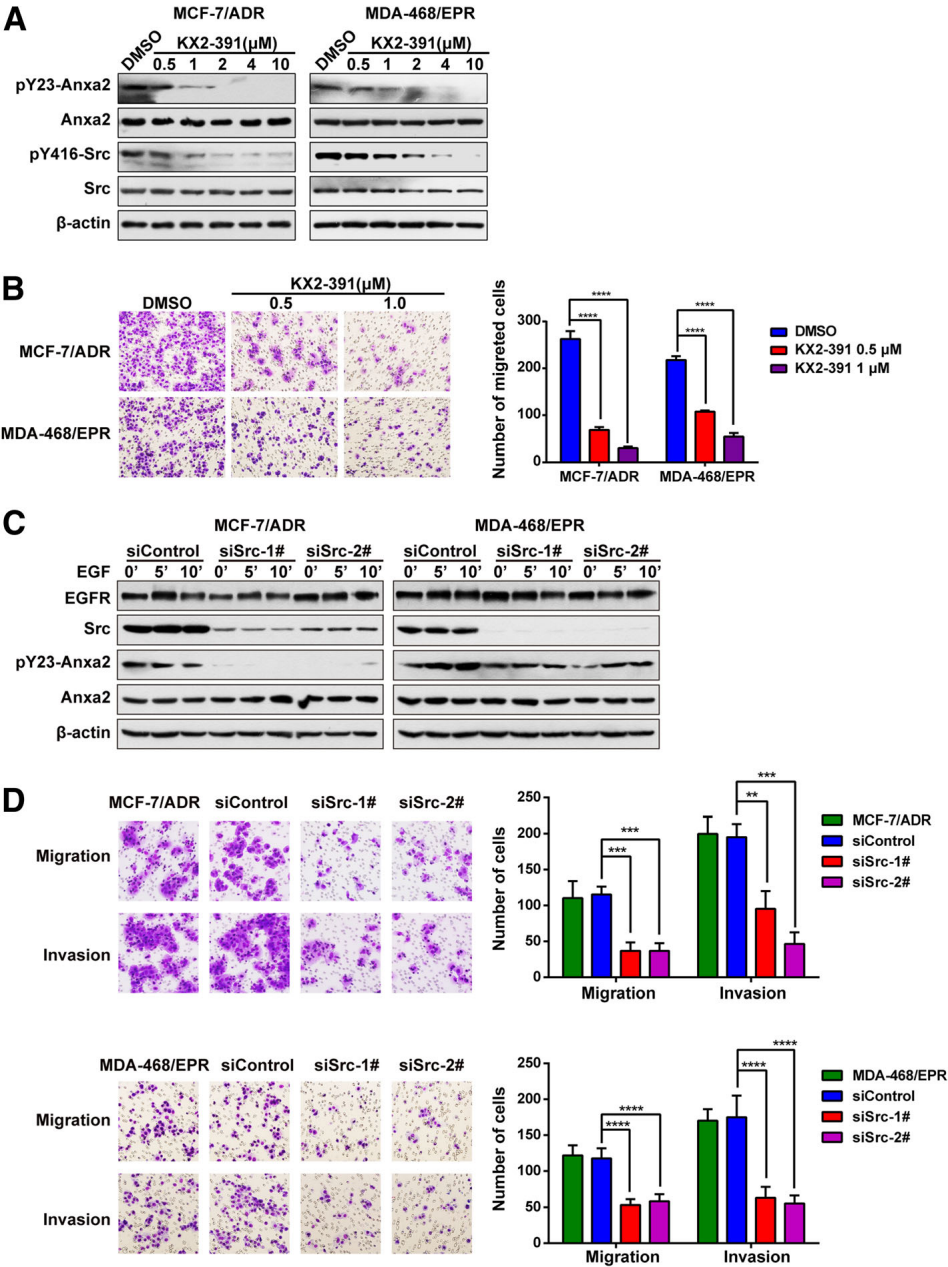
Inhibition of Src kinase blocked Anxa2 phosphorylation and decreased invasiveness of drug-resistant breast cancer cells. **a** Inhibition of Src kinase by using Src kinase inhibitors blocked the phosphorylation of Anxa2 at Tyr23 site in drug-resistant cells. β-actin was used as the loading control. **b** The cell invasion ability was significantly suppressed in Src inhibitor-treated group compared with the control group. Data are shown as mean ± SD; *n* = 6; ****P < 0.0001 compared with DMSO control. **c** Src knockdown evidently inhibited EGF-induced tyrosine phosphorylation of Anxa2 in drug-resistant cells. β-actin was used as the loading control. **d** Knockdown of Src expression inhibited cell migration and invasion ability. The assays were repeated three times. Data are shown as mean ± SD; *n* = 6; **P < 0.01, ***P < 0.001, and ****P < 0.0001 versus control

Src is a well-known upstream kinase of Anxa2 [45–47]. Therefore, to investigate whether the decreased level of pY23-Anxa2 is associated with the declined cell invasion ability in drug-resistant cells, we blocked Src kinase activity in drug-resistant cells by using Src kinase inhibitor KX2-391. As shown in Fig. 2a, the inhibitor efficiently inhibited the phosphorylation of Src at the tyrosine 416 site, indicating the blockage of this kinase activity. Meanwhile, the level of pY23-Anxa2 was remarkably decreased. Figure 2b shows that the cell invasion ability was significantly suppressed in the Src inhibitor-treated group compared with the control group. Moreover, we silenced the expression of Src in two drug-resistant cells by using two different siRNAs, as shown in Fig. 2c. Src expression was significantly downregulated after transfection of two different siRNAs. Moreover, Src knockdown significantly inhibited EGF-induced tyrosine phosphorylation of Anxa2. While the decrease in the level of pY23-Anxa2 after Src knockdown in MDA-MB-468/EPR cells was not as evident as that in MCF-7/ADR cells, this difference may be due in part to the fact that Src may play other roles in MDA-MB-468/EPR cells in addition to modulating Anxa2 phosphorylation or that a fraction of Anxa2 may also be phosphorylated by other unknown kinases. Moreover, transwell assay showed that the migration and invasion abilities in Src knockdown group were significantly decreased compared with that in control cells (Fig. 2d). Collectively, these findings demonstrated that Src kinase is required for Anxa2 tyrosine phosphorylation, and this result is correlated with the invasiveness of drug-resistant cells.

### Anxa2 tyrosine phosphorylation is required for the invasiveness of drug-resistant breast cancer cells

**Fig. 3:**
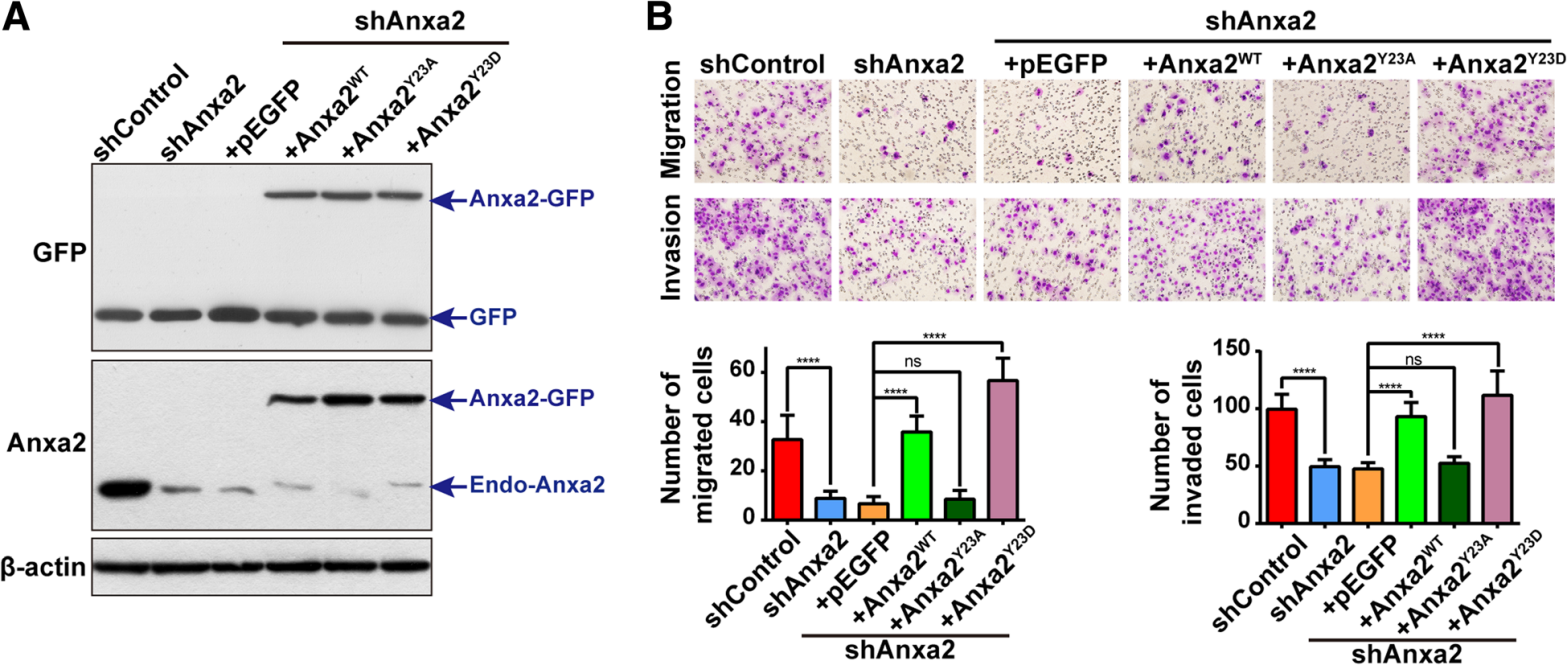
Anxa2 tyrosine phosphorylation is required for the invasiveness of drug-resistant breast cancer cells. **a** The expression of Anxa2 and its mutants, Anxa2^Y23A^ and Anxa2^Y23D^, were effectively rescued in Anxa2-silenced MCF-7/ADR cells as verified by Western blot analysis using anti-Anxa2- or anti-GFP-specific antibodies. β-actin was used as a loading control. **b** The re-expression of Anxa2^WT^ and the phospho-mimicking Anxa2^Y23D^ mutant, not the phospho-deficient Anxa2 ^Y23A^ mutant, in Anxa2-silenced cells rescued the migration and invasion ability. The cell migration and invasion assays were performed by transwell assay. Data are shown as mean ± SD; *n* = 6; ****P < 0.0001 and ^ns^*P* > 0.05 indicate no statistical significance. The statistical results are summarized in the following panel

To determine whether Anxa2 tyrosine phosphorylation is required for the invasiveness of drug-resistant cells, lentivirus expressing GFP-tagged wild-type Anxa2 (Anxa2^WT^) as well as its two mutants, Anxa2^Y23A^ (phospho-deficient mutant) and Anxa2^Y23D^ (phospho-mimicking mutant), were used to infect Anxa2-silenced MCF-7/ADR cells, which were constructed in our previous study [18]. As shown in Fig. 3a, the expression of Anxa2 was effectively rescued in Anxa2-depleted MCF-7/ADR cells, as verified by Western blot analysis using anti-Anxa2 or anti-GFP-specific antibodies. Consistent with previous reports, transwell assays showed that Anxa2 knockdown significantly inhibited the migration and invasion abilities compared with control cells (Fig. 3b). The re-expression of Anxa2^WT^ in Anxa2-silenced MCF-7/ADR cells restored cell migration and invasion abilities, whereas the re-expression of Anxa2 ^Y23A^ mutant or GFP failed to rescue cell motility (Fig. 3b). Moreover, the rescued expression of Anxa2^Y23D^ mutant even showed an increased migration and invasion abilities compared with that of Anxa2^WT^-expressing cells (Fig. 3b). In summary, these data suggested that Anxa2 tyrosine phosphorylation is required for the invasiveness of drug-resistant breast cancer cells.

### Rack1 mediates the binding between Src and Anxa2

**Fig. 4:**
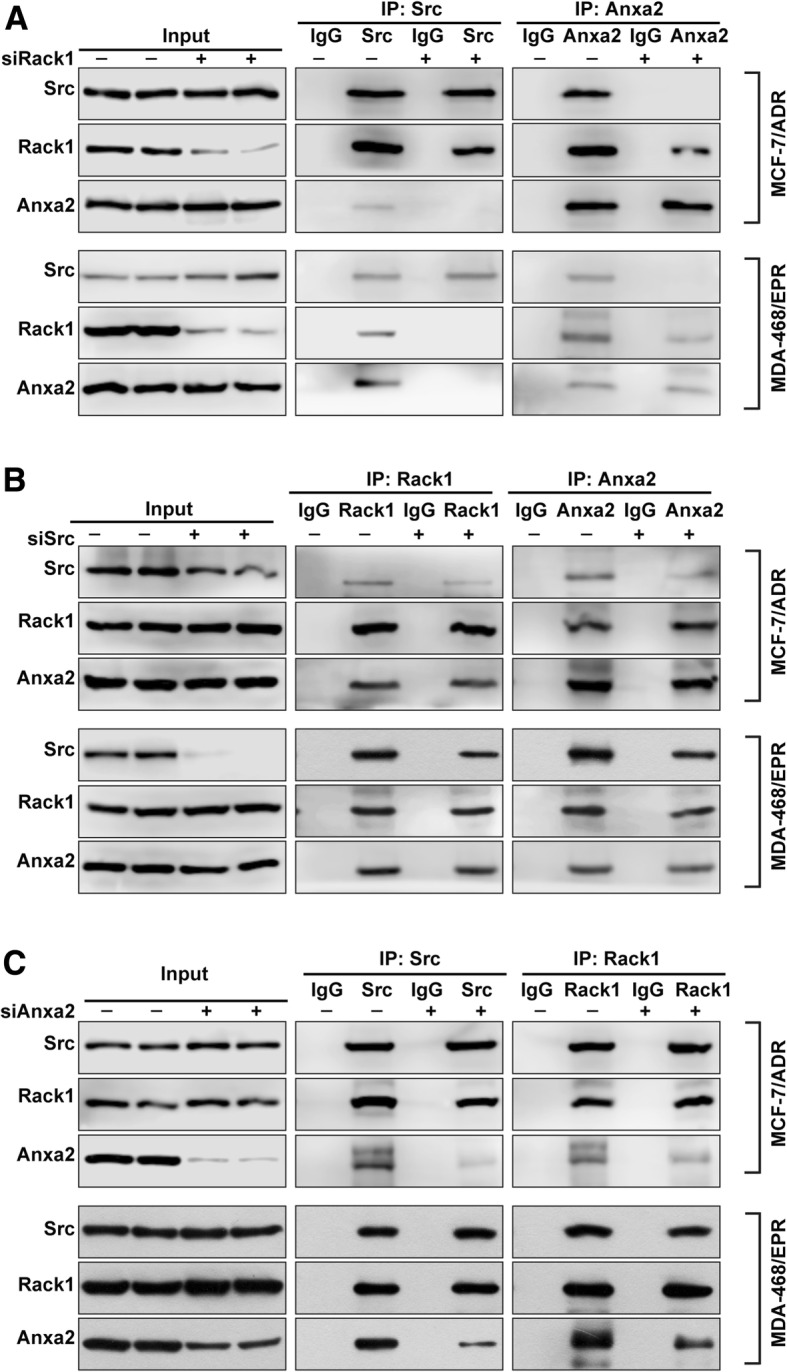
Rack1 mediates the binding between Src to Anxa2. **a** Knockdown of Rack1 expression attenuated the interaction between Src and Anxa2 in two drug-resistant cells. Control or Rack1 knockdown cells were lysed, immunoprecipitated with ant-Src or anti-Anxa2 antibodies, and analyzed by Western blotting with anti-Anxa2, Rack1, or Src antibodies. **b** Silence of Src expression has no significant effect on the binding of Rack1 to Anxa2. Control and Src-silenced cells were lysed, immunoprecipitated with anti-Rack1 or anti-Anxa2 antibodies, and then analyzed by Western blotting using anti-Anxa2, Rack1, or Src antibodies. **c** Anxa2 knockdown has no significant effect on the interaction between Src and Rack1. Control and Anxa2 knockdown cells were lysed and immunoprecipitated with anti-Src or Rack1 antibodies, followed by Western blotting analysis using anti-Anxa2, Rack1, or Src antibodies

We previously showed that the scaffold protein Rack1 forms a complex with Src and Anxa2 in drug-resistant cells [28]. We proposed that Rack1 may mediate the binding between Src and Anxa2, given that Rack1 and Src can bind to Anxa2 and modulate its tyrosine phosphorylation. To test this hypothesis, we first examined the effect of Rack1 knockdown on the binding ability of endogenous Src to Anxa2 in two drug-resistant cells through a Co-IP assay using anti-Src or Anxa2 antibodies. As shown in Fig. 4a, Rack1 knockdown conferred no evident effect on the expression levels of Src and Anxa2, whereas the interaction between Src and Anxa2 in two Rack1-silenced cells was notably attenuated compared with that in control cells (Fig. 4a). Consistently, a reciprocal Co-IP assay using anti-Anxa2 antibodies also showed that the binding ability of Anxa2 to Src was decreased in two Rack1-knockdown cells compared with control cells. Therefore, these data suggest that Rack1 is required for the binding of Src to Anxa2. In addition, to investigate whether Src is necessary for the interaction between Rack1 and Anxa2, we silenced Src expression in two drug-resistant cells by using siRNAs and performed Co-IP assay with anti-Rack1 or Anxa2 antibodies. As shown in Fig. 4b, the expression levels of Rack1 and Anxa2 were unchanged after knockdown of Src in the two drug-resistant cells. The amount of Anxa2 co-precipitated by anti-Rack1 antibodies in cell lysates from control cells was similar to that from Src knockdown cells (Fig. 4b). Similarly, a reciprocal Co-IP assay with anti-Anxa2-specific antibodies also confirmed that the binding ability of Anxa2 to Rack1 was not altered in the absence of Src (Fig. 4b). These results suggest that the binding of Anxa2 to Rack1 is unaffected by Src knockdown. Moreover, we further determined whether Anxa2 is necessary for the interaction of Rack1 and Src. As shown in Fig. 4c, Anxa2 knockdown did not affect the expression levels of Src and Rack1. Co-IP assay with anti-Src antibodies showed that the amount of Rack1 bound to Src was the same in the control and Anxa2-silenced cells (Fig. 4c). Consistently, anti-Rack1 antibodies also co-precipitated a similar amount of Src, indicating that the interaction of Rack1 and Src was independent of the presence of Anxa2. These results suggested that Rack1 functions as a scaffold protein and mediates the interaction between Src and Anxa2.

### Rescued expression of Rack1^WT^, not the Src binding-deficient Rack1^Y246F^ mutant, restored Anxa2 phosphorylation and invasion ability in drug-resistant cancer cells

**Fig. 5:**
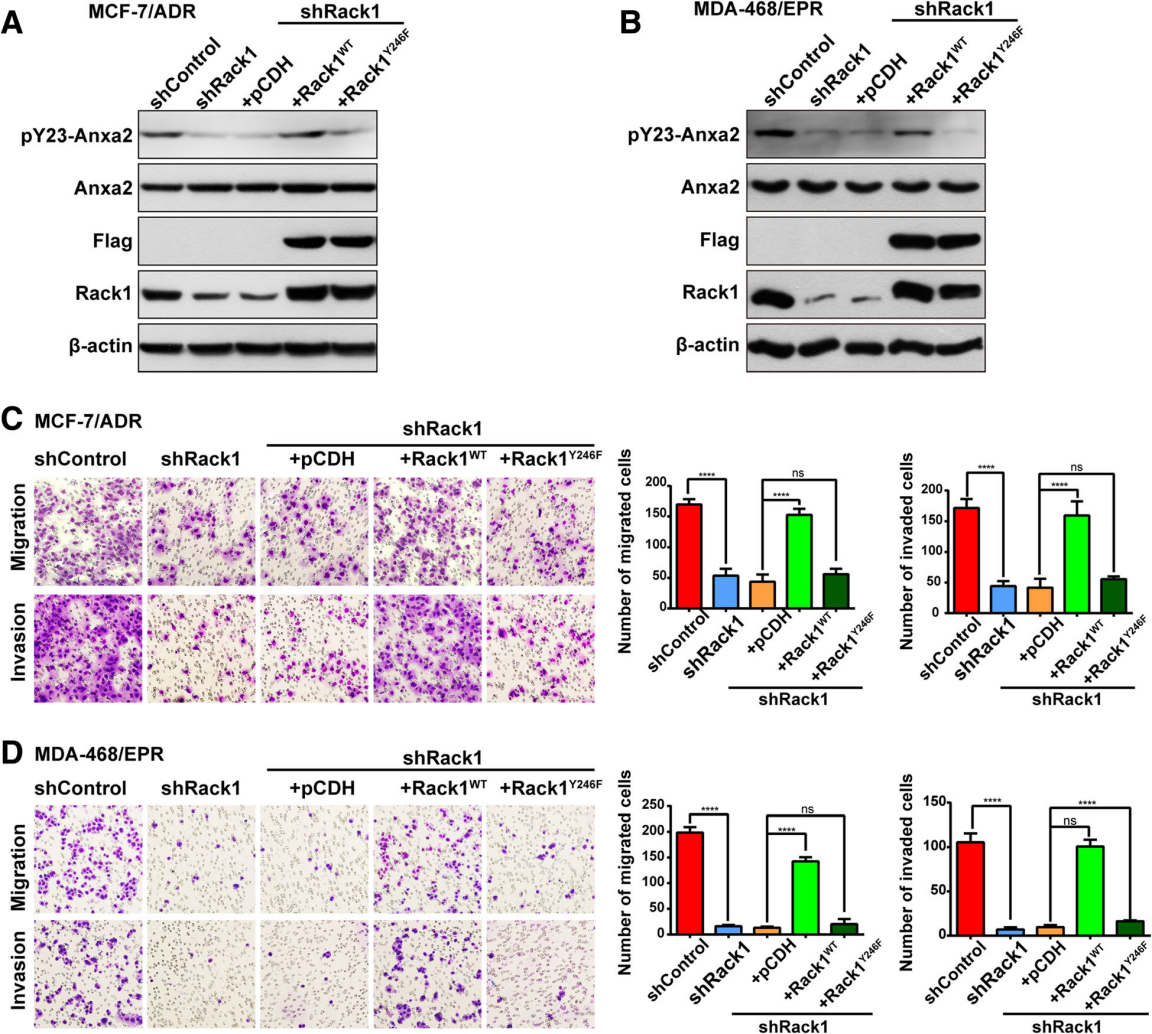
Re-expression of Rack1^WT^, not Rack1^Y246F^, rescued Anxa2 phosphorylation and invasion ability in drug-resistant cancer cells. **a**, **b** Rescued expression of Rack1^WT^, not the Src binding-deficient Rack1^Y246F^ mutant, recovered Anxa2 phosphorylation in MCF-7/ADR cells (**a**) and in MDA-MB-468/EPR cells (**b**). Rack1 expression was stably silenced by using shRNA targeting its noncoding region. Then, the cells were infected with lentivirus expressing flag-tagged Rack1^WT^ or Rack1^Y246F^ and selected by using hygromycin B. The cell lysates were analyzed by Western blotting with anti-Rack1, Flag, Anxa2, and phospho-Anxa2 antibodies. **c**, **d** Re-expression of Rack1^WT^, not Rack1^Y246F^, recovered cell migration and invasion abilities. Transwell assay was used to analyze the migration and invasion abilities in MCF-7/ADR cells (**c**) and in MDA-MB-468/EPR cells (**d**). Representative images were captured at 200×. Statistical analysis was performed by one-way ANOVA. Statistical results were exhibited in the following panel. ****P < 0.0001 and ^ns^*P* > 0.05 indicate no statistical significance

The findings prompted us to further confirm whether Rack1 modulates Anxa2 phosphorylation and cell invasion through its binding to Src. To validate this speculation, Rack1 stable knockdown in two drug-resistant cells was established by using a n shRNA, specifically targeting to its noncoding region. Then, we rescued Rack1 expression in Rack1-silenced cells through infection of lentivirus expressing Flag-tagged Rack1^WT^ and Src binding-deficient Rack1^Y246F^ mutant. As shown in Fig. 5a and b, Rack1 expression was effectively rescued in Rack1-silenced cells to levels similar to those in endogenous proteins, as verified by Western blot analysis using anti-Rack1 or anti-Flag antibodies. Re-expression of Rack1^WT^ in Rack1-silenced cells rescued Anxa2 tyrosine phosphorylation compared with that of control cells. However, rescuing with the Rack1^Y246F^ mutant failed to recover Anxa2 phosphorylation. Therefore, these results suggested that Anxa2 tyrosine phosphorylation was regulated by Src in a Rack1-dependent manner. Next, we compared the cell migration and invasion ability among these cells. As shown in Additional file 2: Figure S3, wound healing assay showed that re-expression of Rack1^WT^ significantly increased cell migration ability. On the contrary, re-expressing Rack1^Y246F^ failed to rescue the cell migration defects caused by Rack1 knockdown. Similarly, transwell-based assay showed that rescuing with Rack1^WT^, but not with Rack1^Y246F^ mutant, recovered cell invasion ability (Fig. 5c, d). These data suggested that Rack1 mediates tyrosine phosphorylation of Anxa2 by Src and promotes invasion in drug-resistant breast cancer cells.

### Increased expression of Anxa2^WT^ or Anxa2^Y23D^ in Rack1-silenced cells recovered cell invasion ability

**Fig. 6:**
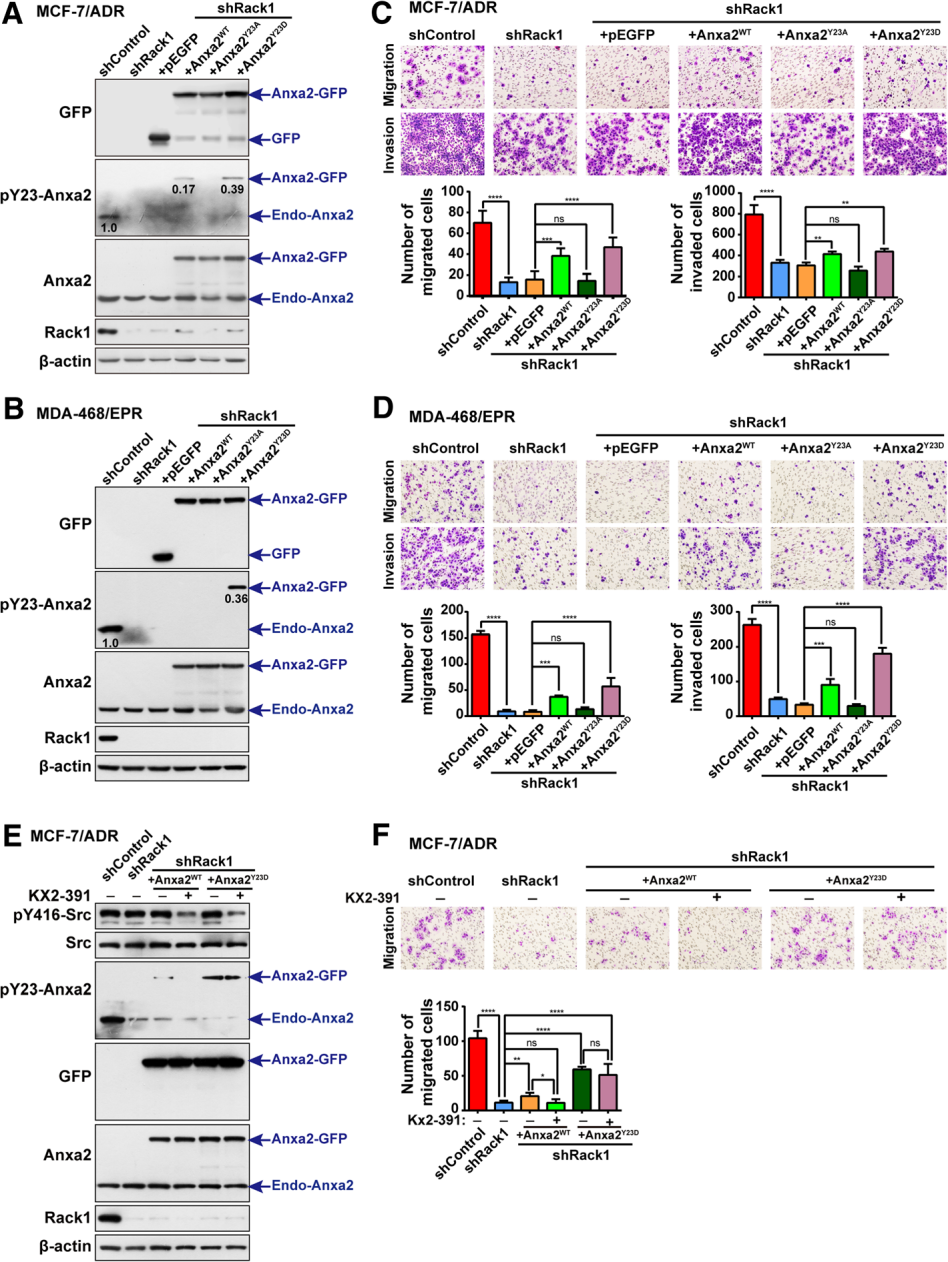
Increased expression of Anxa2^WT^ or Anxa2^Y23D^ in Rack1-silenced cells recovered cell invasion ability. **a**, **b** Increased expression of Anxa2^WT^, Anxa2^Y23A^, and Anxa2^Y23D^ in Rack1 stably silenced MCF-7/ADR cells (**a**) and MDA-MB-468/EPR cells (**b**). Lentivirus expressing Anxa2^WT^, as well as its two mutants, were used to infect Rack1 stably silenced cells, in which the expression of Rack1 has been stably transfected with an shRNA targeting its noncoding region. Then, the cells were lysed and analyzed by Western blotting using anti-Anxa2, anti-GFP, and anti-Rack1 antibodies. β-actin was used as a loading control. **c**, **d** Transwell assays showed that overexpression of Anxa2^WT^ or Anxa2^Y23D^, not Anxa2^Y23A^, in Rack1-silenced MCF-7/ADR (**c**) and MDA-MB-468/EPR cells (**d**) partially rescued the cell migration and invasion abilities. Data are shown as mean ± SD; *n* = 6. Statistical analysis was performed by one-way ANOVA. *P < 0.05, **P < 0.01, ***P < 0.001, ****P < 0.0001, and ^ns^*P* > 0.05 indicate no statistical significance. **e** Src inhibitor KX2-391 efficiently inhibited the expression of pY23-Anxa2 in Anxa2^WT^-expressing cells, while had no significant effect on the level of pY23-Anxa2 in Anxa2^Y23D^-expressing cells as detected by Western blotting. **f** Transwell assay showed that the migration ability in Anxa2^WT^-expressing cells can be quenched by Src inhibitor, while Src inhibition has little effect on the migration ability in Anxa2^Y23D^-expressing cells. Data are shown as mean ± SD; *n* = 6. Statistical analysis was performed by one-way ANOVA. *P < 0.05, **P < 0.01, ****P < 0.0001, and ^ns^*P* > 0.05 indicate no statistical significance

To further determine whether the decreased invasiveness of Rack1-silenced cells occurred mechanically through inhibiting Anxa2 phosphorylation, we introduced GFP-tagged Anxa2^WT^ or its two mutants into Rack1 stable knockdown cells using lentivirus. As shown in Fig. 6a and b, the expression level of Anxa2 and its mutants were successfully increased in Rack1-silenced cells, as measured by Western blot analysis using anti-Anxa2 or anti-GFP antibodies. The pY23-Anxa2 was slightly higher in Anxa2^WT^-expressing MCF-7/ADR cells than in the Rack1 knockdown cells, this is partly due to the total level (endogenous Anxa2 and Anxa2-GFP) of Anxa2 was higher in Anxa2^WT^-expressing cells than in the Rack1 knockdown cells, and the presence of less active Src may still phosphorylate a small fraction of Anxa2 in these cells, while the level of pY23-Anxa2 was apparently lower in Anxa2^WT^-expressing cells than in control and Anxa2^Y23D^-expressing cells. Then, we performed wound healing assay to analyze the cell migration ability. As shown in Additional file 2: Figure S4, the increased expression of Anxa2^WT^ or Anxa2^Y23D^ significantly enhanced cell migration ability compared with that of GFP-expressing cells, whereas the increased expression of Anxa2^Y23A^ mutant failed to rescue the cell migration ability. Consistently, transwell-based assays showed that elevated expression of Anxa2^WT^ or Anxa2^Y23D^ in Rack1-knockdown cells notably recovered cell invasion ability. On the contrary, increased expression of Anxa2^Y23A^ mutant failed to restore the migration and invasion abilities (Fig. 6c, d), although the rescued cell migration/invasion ability in Anxa2^WT^- or Anxa2^Y23D^-expressing cells cannot reach to that of wild-type cells. Moreover, we further treated Anxa2^WT^- and Anxa2^Y23D^-expressing MCF-7/ADR cells with Src inhibitor KX2-391. As shown in Fig. 6e, KX2-391 efficiently inhibited the expression of pY23-Anxa2 in Anxa2^WT^-expressing cells, while the level of pY23-Anxa2 in Anxa2^Y23D^-expressing cells was not affected by Src kinase inhibitor. In addition, transwell-based assay showed that the migration ability of Anxa2^WT^-expressing cells can be quenched by Src inhibitor, while Src inhibition has little effect on the migration ability in phospho-mimicking Anxa2^Y23D^-expressing cells (Fig. 6f). These results suggested that the decreased cell migration and invasion abilities in Rack1-silenced cells were attributed, at least in part, to the inhibition of Anxa2 tyrosine phosphorylation.

### Rack1 is critical for drug-resistant breast cancer cell metastasis in vivo

**Fig. 7:**
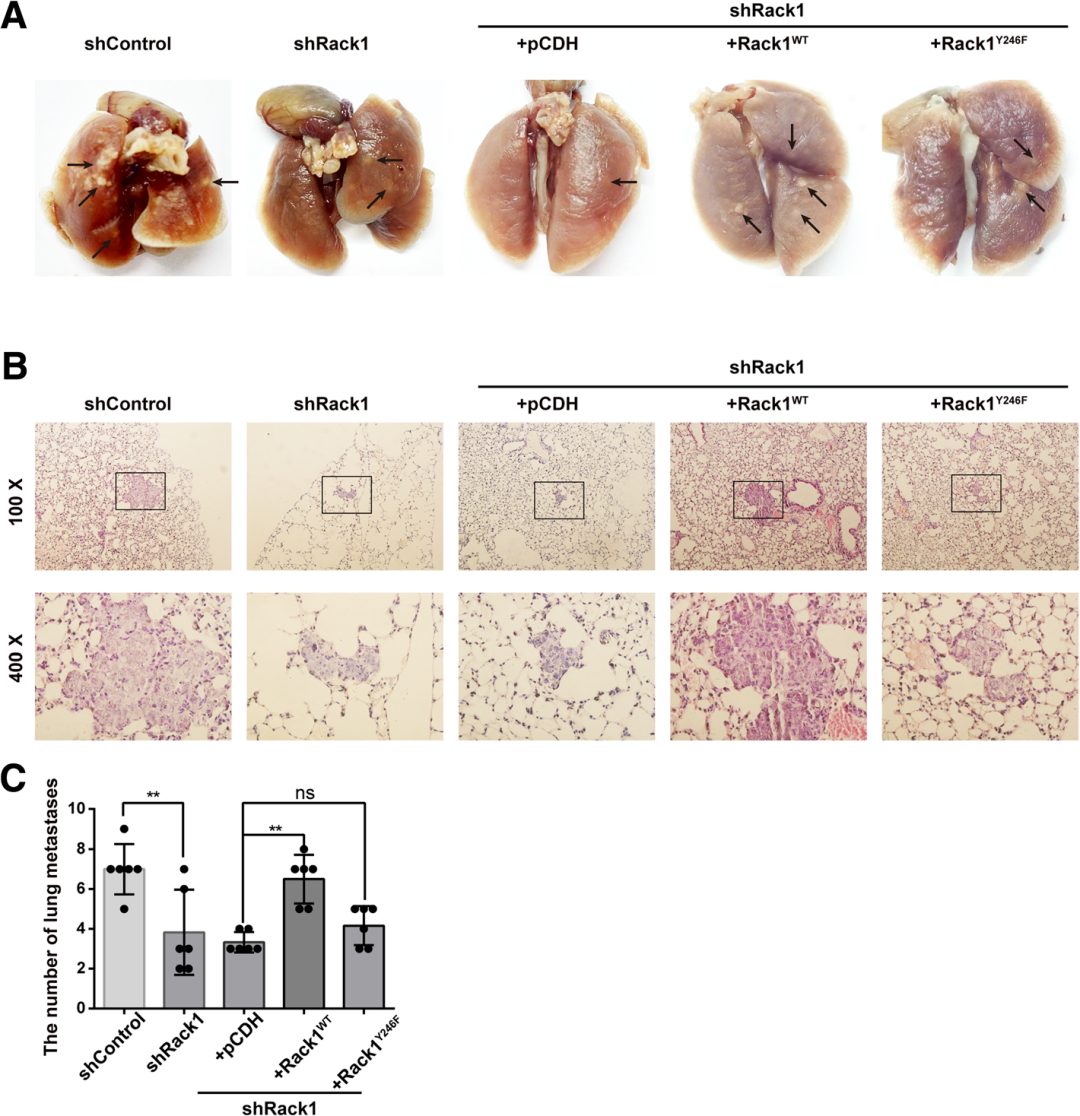
Rack1 is critical for drug-resistant breast cancer cell metastasis in vivo. **a** Rescued with Rack1^WT^ group displayed more metastatic foci on the mice lung surface than that in control and Rack1^Y246F^-rescued group. **b**, **c** Hematoxylin and eosin staining showed that rescued with Rack1^WT^, not Rack1^Y246F^, in Rack1-silenced cells increased the number of micrometastases in the lung compared with that of the control group. Data are shown as mean ± SD; *n* = 8. Statistical analysis was performed by one-way ANOVA. **P < 0.01 and ^ns^*P* > 0.05 indicate no significance

To further investigate the functions of Rack1 on the metastatic potential of drug-resistant breast cancer cells in vivo, we used pulmonary metastasis model in NOD-SCID mice via tail vain injection of MCF-7/ADR cells, control, and the Rack1 stable knockdown cells, as well as the Rack1^WT^- and Rack1^Y246F^-rescued cells. Three months after injection, apparent reduction of tumor metastatic foci was shown in the lung surface of Rack1-silenced group compared with the control group (Fig. 7a). In addition, the lung surface rescued with the Rack1^WT^ group displayed more metastatic foci than that in control and Rack1^Y246F^-rescued group (Fig. 7a). Moreover, we stained mice lung tissue sections with hematoxylin and eosin to confirm the metastatic foci. As shown in Fig. 7b and c, the size and the number of tumor foci in the lung were significantly decreased in Rack1-silenced group compared with those in the control group. Furthermore, the rescued expression of Rack1^WT^, but not Rack1^Y246F^ mutant, in Rack1-silenced cells increased the number of metastases in the lung compared with that of the control group. Collectively, these data demonstrated that Rack1 is critical for the metastatic potential of drug-resistant breast cancer cells in vivo.

## Discussion

A high level of Anxa2 in cancer tissues is correlated with a highly aggressive phenotype [15, 46, 48–51]. Increased Anxa2 expression has been shown to be specific in many drug-resistant cancer cells [2, 16–20]. Moreover, recent studies have shown that Anxa2 is a key protein that links drug resistance and cancer metastasis [2, 12, 15]. The functional activity of Anxa2 has been shown to be regulated by tyrosine phosphorylation at the Tyr23 site [13]. Nevertheless, the accurate molecular mechanisms underlying the regulation of Anxa2 tyrosine phosphorylation and whether phosphorylation is necessary for the enhanced invasive phenotype of drug-resistant cells remain unknown. In this study, we demonstrated that Anxa2 Tyr23 phosphorylation is required for MDR breast cancer invasion and metastasis. Anxa2 functions downstream of Rack1/Src complex, and Rack1 is required for EGF-induced tyrosine phosphorylation of Anxa2, as well as the migration and invasion abilities in drug-resistant cells. Moreover, we provided evidence that Rack1 acts as a signal hub and mediates the interaction between Src and Anxa2, thereby facilitating Anxa2 phosphorylation by Src kinase. Hence, our findings suggest a new role of Rack1 in regulating Anxa2 tyrosine phosphorylation.

Elevated Anxa2 expression seems a common event when cancer cells acquire drug resistance (Additional file 2: Figure S5 A and C) [2, 16–20, 45, 52, 53]. Anxa2 overexpression promotes chemoresistance in non-small cell lung cancer, pancreatic cancer, neuroblastoma, and hepatocarcinoma [16, 17, 54, 55]. Moreover, Anxa2 confers increased invasiveness in breast cancer (Additional file 2: Figure S5 B and C) [18, 56, 57]; Anxa2 knockdown also inhibits migration and invasion in nasopharyngeal carcinoma [21, 22]. However, whether Anxa2 expression per se or its phosphorylated form confers the aggressive phenotype of drug-resistant cells remains unclear. We observed that the reduction of phosphorylated Anxa2 was associated with decreased invasiveness in MDR breast cancer cells. In addition, re-expression of phospho-mimicking Anxa2^Y23D^, but not the phospho-deficient Anxa2^Y23A^ mutant, in Anxa2-silenced cells restored cell invasive potential. Expression of Anxa2^Y23D^ even showed an increased invasion ability compared with that of Anxa2^WT^-expressing cells. These results suggested a functional association between Anxa2 Tyr23 phosphorylation and invasive behavior in resistant cells. Several studies have shown that chemotherapeutic agents can enhance phosphorylation of Anxa2 in cancer cells [12, 27], and anti-cancer drugs have been shown to induce highly aggressive phenotypes in resistant cells [9, 11, 58]. Additionally, phosphorylated Anxa2 confers resistance in several cancer cells [45, 59]. Consistent with other studies, we have proven that Anxa2 Tyr23 phosphorylation is critical in the invasion and metastasis of many types of cancer [48, 56, 60]. These results indicated that increased Anxa2 Tyr23 phosphorylation can be a mechanism for drug-resistant cells to enhance its migration and invasion abilities.

To date, the precise mechanism regulating Anxa2 Tyr23 phosphorylation remains largely unknown. We previously showed that the scaffold protein Rack1 binds Anxa2 and regulates Adriamycin-induced phosphorylation of Anxa2 [28]. Rack1 knockdown inhibits basal level and EGF-induced phosphorylation of Anxa2 in drug-resistant cells, thus suggesting that Rack1 is a key molecule that modulates Anxa2 phosphorylation. Src is the major kinase that phosphorylates Anxa2 in many types of cells, and the blockage of Src activity with inhibitors or siRNAs inhibits phosphorylated Anxa2 in two resistant cells. Rack1 is known as a signaling bridge mediating protein–protein interaction [30]. These data raised a possibility that Rack1 may nucleate a complex with Src and Anxa2, thereby facilitating Anxa2 phosphorylation by Src. Rack1 knockdown notably attenuated the binding of Src to Anxa2, whereas silencing the expression of Anxa2 or Src does not affect their interaction with Rack1, demonstrating that Rack1 mediates the interaction between Src and Anxa2. Consistently, our finding showed that re-expression of Rack1^WT^, not the Src binding-deficient Rack1^Y246F^ mutant, in Rack1-silenced cells restored Anxa2 phosphorylation; this finding further confirmed this hypothesis. Taken together, our results suggest that Rack1 modulates Anxa2 tyrosine phosphorylation through a Src-dependent manner. To our knowledge, this study is the first to demonstrate the regulatory mechanism of Anxa2 phosphorylation by Rack1.

Deregulated expression of Rack1 has been reported in many types of carcinoma, and the function of Rack1 on cancer invasion and metastasis appears cancer type- and cell context-specific [29, 30]. Rack1 inhibits the invasion ability of colon cancer cells [33]. Moreover, Rack1 negatively regulates the invasiveness and metastasis of cancer cells in gastric cancer [32, 34]; On the contrary, Rack1 promotes invasion and metastasis of prostate cancer and hepatocellular carcinoma cells [61, 62]; Consistently, knockdown of Rack1 reduces invasion in melanoma, oral squamous carcinoma cells, and lung cancer cells [35, 36, 38, 63]. In this study, silencing Rack1 in drug-resistant cells inhibited invasion ability in vitro and metastasis potential in vivo. Thus, our results support the tumor-promoter role of Rack1 in breast cancer. These data were in line with previous studies, showing that Rack1 promotes aggressive behavior in breast cancer [64, 65]. Interestingly, recent studies have shown that Rack1 also confers resistance to cancer. Rack1 elevation enhances chemoresistance in leukemia, hepatocellular carcinoma, and esophageal cancer [39, 41, 43]; Rack1 overexpression promotes targeted therapy resistance in gastrointestinal stromal tumor [42]. These findings indicate that Rack1 might also be a critical hub involved in the crosstalk between drug resistance and invasion/metastasis. We proposed that Anxa2 functions downstream of Rack1 to promote the aggressive behavior in drug-resistant cells given that Rack1 binds and regulates Anxa2 phosphorylation, and Anxa2 is a key protein causing drug resistance and enhancement of cancer metastasis [2]. In support of this hypothesis, exogenous expression of Anxa2^WT^ or Anxa2^Y23D^ instead of Anxa2^Y23A^ mutant notably rescued the migration and invasion defects in Rack1-silenced cells. These data suggest that Anxa2 mediates the biological function of Rack1/Src in drug-resistant breast cancer cells.

Although we have demonstrated the critical role of the Rack1/Src/Anxa2 complex in promoting aggressive behavior of drug-resistant cells, the accurate molecular mechanisms downstream of the complex remain to be determined. We have reported that pY23-Anxa2 binds to and enhances STAT3 activation and promotes invasion and metastasis of breast cancer cells [56]. Consistently, Anxa2 associates with STAT3 and confers the aggressive behavior in colorectal cancer [46, 66]. Interestingly, Rack1 is also involved in the activation of STAT3 in several cancer cells [36, 67]. Src is also a well-known upstream kinase of STAT3, and enhanced STAT3 activation confers invasiveness of drug-resistant breast cancer cells [8]. Therefore, these findings suggest that a possible downstream pathway of the Rack1/Src/Anxa2 complex may involve STAT3 signaling. In addition, another mechanism by which this complex promotes tumor invasion and metastasis may be through regulation of actin remodeling, which is essential for the migration and invasion of cancer cells. It has been reported that pY23-Anxa2 is involved in the regulation of Rho-mediated actin rearrangement [51, 60, 68, 69]. Interestingly, Rack1 also interacts with Rho and activates RhoA/Rho kinase pathway to enhance breast cancer metastasis [64]. Moreover, several studies have shown that pY23-Anxa2 promotes cancer cell invasion and metastasis through enhancing epithelial-mesenchymal transition (EMT) [46, 60, 70]. Likewise, Rack1 has also been reported to regulate EMT and contribute cancer metastasis [71, 72]. Drug-resistant cells are always associated with EMT phenotype [73–75]. Hence, EMT may also be a possible mechanism for Rack1/Src/Anxa2 complex to promote the malignant behavior of drug-resistant cells. In future studies, it will be interesting to delineate the detailed mechanism by which this complex promotes invasion and metastasis of drug-resistant cells.

## Conclusion

In summary, our study demonstrated a pivotal role of Rack1 involved in the crosstalk between drug resistance and cancer invasion/metastasis. Rack1 is required for the invasive and metastatic potential of MDR breast cancer cells through mediating the binding of Anxa2 to Src, thereby facilitating Anxa2 phosphorylation by Src kinase. The interaction between Anxa2 and Rack1/Src is responsible for the association between MDR and invasive potential in breast cancer cells. Thus, our findings provide novel insights on the mechanism underlying the functional linkage between drug resistance and cancer aggressive.

## Additional files

Additional file 1: Supplementary methods: Immunofluorescence staining and Apoptosis assay. (PDF 90 kb)

Additional file 2: **Figure S1.** The expression of EGFR was higher in MCF-7/ADR cells than in MDA-MB-468/EPR cells. (A) The expression of EGFR in MCF-7/ADR cells were higher than that in MDA-MB-468/EPR cells. (B) The expression of EGFR in two drug-resistant cells mainly located in the cell membrane. **Figure S2.** Knockdown of Rack1 had no significant effect on apoptosis in drug-resistant cancer cells. (A and B) Knockdown of Rack1 in two drug-resistant cells had no significant effect on cell death compared with that in control cells. The proportion of apoptotic cells at early stage (PI^−^/Annexin V^+^) and late stage (PI^+^/Annexin V^+^) was shown; mean ± SD, *n* = 3, ns means no statistical difference. **Figure S3.** Re-expression of Rack1^WT^, not Rack1^Y246F^, rescued migration ability in drug-resistant cancer cells. (A) Re-expression of Rack1^WT^, not Rack1^Y246F^, rescued cell migration ability in MCF-7/ADR cells. (B) The relative cell migration distance was quantified and plotted in the lower panel. Data are shown as mean ± SD; *n* = 6. ***P < 0.001 and ns indicates no statistical significance. **Figure S4.** Increased expression of Anxa2^WT^ or Anxa2^Y23D^ in Rack1-silenced cells recovered cell migration ability. (A and B) Overexpression of Anxa2^WT^ or Anxa2^Y23D^, not Anxa2^Y23A^, partially rescued the cell migration ability in Rack1-silenced MCF-7/ADR cells. Data are shown as mean ± SD; *n* = 6. ****P < 0.0001 and **P < 0.01. **Figure S5.** Silencing of Anxa2 expression attenuates migration ability in breast cancer cells. (A) The expression of Anxa2 and pY23-Anxa2 in MCF-7/ADR cells were elevated compared with that in MCF-7 cells. (B and C) Knockdown of Anxa2 in MCF-7 and MCF-7/ADR cells decreased migration ability. Data were displayed as mean ± SD; *n* = 6. *P < 0.05, ****P < 0.0001 versus shControl. (PDF 814 kb)

## Abbreviations

EGF: Epidermal growth factor
MDR: Multidrug resistant
PCR: Polymerase chain reaction
PDGF: Platelet-derived growth factor
Rack1: Receptor for activated protein C kinase 1
