# Supplementary material for: Rack1 mediates tyrosine phosphorylation of Anxa2 by Src and promotes invasion and metastasis in drug-resistant breast cancer cells
Source: Breast Cancer Res. 2019 May 22;21:66. doi: 10.1186/s13058-019-1147-7 (PMC6530024; doi:10.1186/s13058-019-1147-7)
Supplement: Supplementary file 1 — Supplementary methods: Immunofluorescence staining and Apoptosis assay. (PDF 90 kb) [file 13058_2019_1147_MOESM1_ESM.pdf]

Supplementary methods:

Immunofluorescence staining

Immunofluorescence assay was performed as described previously [1]. Briefly, cells were trypsinized and seeded at 3 × 10^4 cells per well into 12-well plate pre-placed with glass coverslips and cultured for 24 h. Then the cells were washed with PBS, fixed with 4% paraformaldehyde for 10 min, and permeabilized with methanol for 10 min at -20 °C. Afterwards, the cells were blocked with 3% BSA in PBS for 1 h at room temperature, incubated with primary antibodies against EGFR (# 4267, Cell Signaling Technology, 1:100, Rabbit) at 4 °C overnight, and followed by incubation with Alexa Fluor 488-conjugated goat anti-rabbit secondary antibodies for 1 h at room temperature. The nuclei were stained with 1.0 ng/mL of DAPI for 10 min at room temperature. The coverslips were mounted with Mowiol-based anti-quenching medium and imaged by laser scanning confocal microscope (Leica TCS SP5).

Apoptosis assay

Apoptosis assay was determined by flow cytometry using Annexin V-FITC /PI staining kit (#CA1020, Solarbio, Beijing, China). Briefly, control and Rack1 knockdown cells were harvested by trypsinization, and washed with cold PBS thrice. Afterwards, the cells were resuspended in binding buffer at a density of 1 × 10^6 cells/mL, then 100 μL of the cell suspension was transferred to a tube and incubated with 5 μL of Annexin V-FITC antibodies for 10 min at room temperature in the dark. The cells were then incubated with 5 μL of Propidium Iodide staining solution for 5 min at room temperature in darkness. Afterwards, each tube of cells was mixed with 200 μL of PBS and immediately analyzed by flow cytometry.

[1] Zhang F, Zhang H, Wang Z, et al. P-glycoprotein associates with Anxa2 and promotes invasion in multidrug resistant breast cancer cells[J]. Biochem Pharmacol, 2014, 87(2): 292-302.
