# Supplementary material for: Rack1 mediates tyrosine phosphorylation of Anxa2 by Src and promotes invasion and metastasis in drug-resistant breast cancer cells
Source: Breast Cancer Res. 2019 May 22;21:66. doi: 10.1186/s13058-019-1147-7 (PMC6530024; doi:10.1186/s13058-019-1147-7)
Supplement: Supplementary file 2 — Figure S1. The expression of EGFR was higher in MCF-7/ADR cells than in MDA-MB-468/EPR cells. (A) The expression of EGFR in MCF-7/ADR cells were higher than that in MDA-MB-468/EPR cells. (B) The expression of EGFR in two drug-resistant cells mainly located in the cell membrane. Figure S2. Knockdown of Rack1 had no significant effect on apoptosis in drug-resistant cancer cells. (A and B) Knockdown of Rack1 in two drug-resistant cells had no significant effect on cell death compared with that in control cells. The proportion of apoptotic cells at early stage (PI−/Annexin V+) and late stage (PI+/Annexin V+) was shown; mean ± SD, n = 3, ns means no statistical difference. Figure S3. Re-expression of Rack1WT, not Rack1Y246F, rescued migration ability in drug-resistant cancer cells. (A) Re-expression of Rack1WT, not Rack1Y246F, rescued cell migration ability in MCF-7/ADR cells. (B) The relative cell migration distance was quantified and plotted in the lower panel. Data are shown as mean ± SD; n = 6. ***P < 0.001 and ns indicates no statistical significance. Figure S4. Increased expression of Anxa2WT or Anxa2Y23D in Rack1-silenced cells recovered cell migration ability. (A and B) Overexpression of Anxa2WT or Anxa2Y23D, not Anxa2Y23A, partially rescued the cell migration ability in Rack1-silenced MCF-7/ADR cells. Data are shown as mean ± SD; n = 6. ****P < 0.0001 and **P < 0.01. Figure S5. Silencing of Anxa2 expression attenuates migration ability in breast cancer cells. (A) The expression of Anxa2 and pY23-Anxa2 in MCF-7/ADR cells were elevated compared with that in MCF-7 cells. (B and C) Knockdown of Anxa2 in MCF-7 and MCF-7/ADR cells decreased migration ability. Data were displayed as mean ± SD; n = 6. *P < 0.05, ****P < 0.0001 versus shControl. (PDF 814 kb) [file 13058_2019_1147_MOESM2_ESM.pdf]

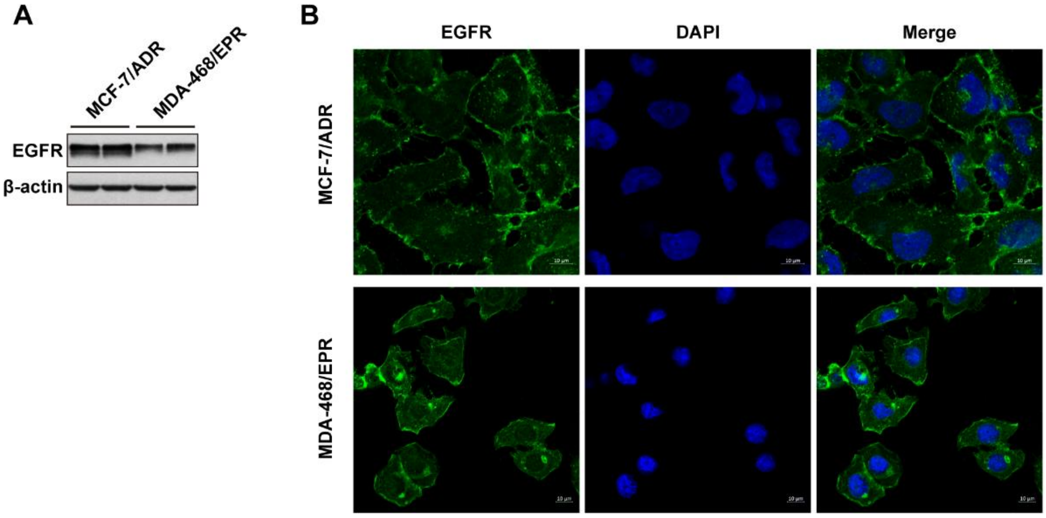

Figure S1. The expression of EGFR was higher in MCF-7/ADR cells than in MDA-MB-468/EPR cells. (A) Western blotting analysis showed that the expression of EGFR in MCF-7/ADR cells were higher than that in MDA-MB-468/EPR cells. (B) Confocal immunofluorescence microscopy analysis showed that the expression of EGFR in two drug-resistant cells mainly located in the cell membrane.

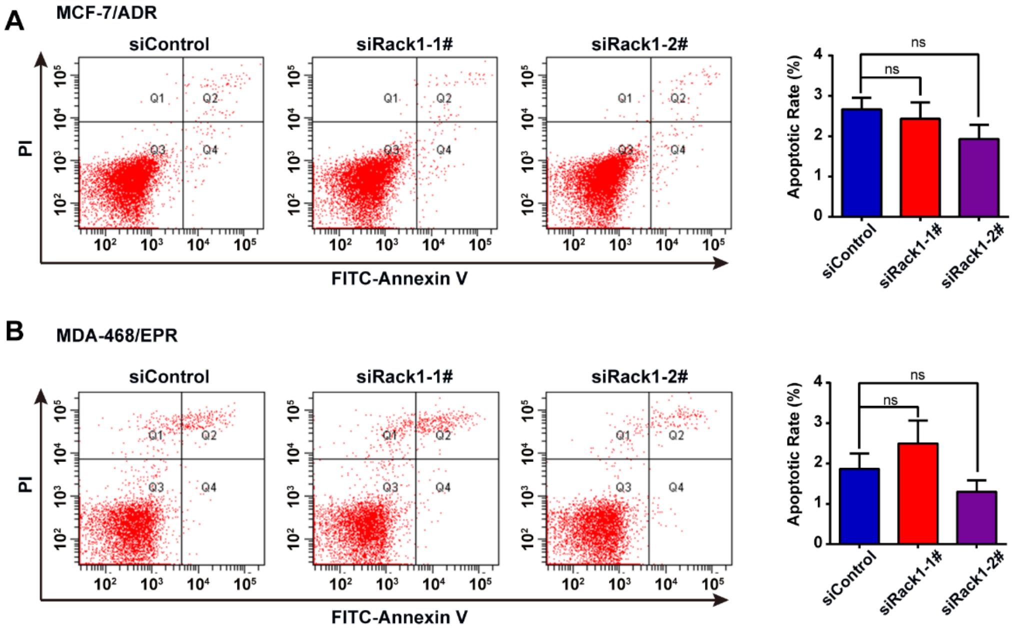

Figure S2. Silencing the expression of Rack1 had no significant effect on apoptosis in drug-resistant cancer cells. (A and B) Flow cytometry based apoptotic assay showed that the knockdown of Rack1 in two drug-resistant cells had no significant effect on cell death compared with that in control cells. The proportion of apoptotic cells at early stage (PI^-/Annexin V^+) and late stage (PI^+/Annexin V^+) was shown; mean ± SD, n = 3, $^{ns}P > 0.05$ versus siControl, ns means no statistical difference.

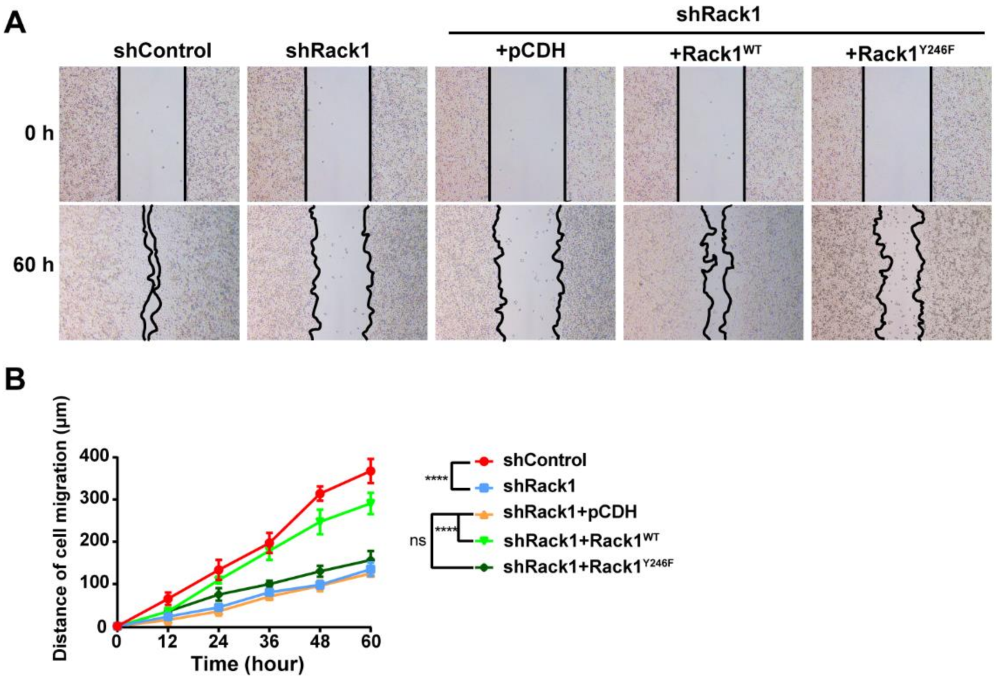

Figure S3. Re-expression of Rack1^{WT}, not Rack1^{Y246F}, rescued migration ability in drug-resistant cancer cells. (A) Wound healing assay showed that re-expression of Rack1^{WT}, but not Rack1^{Y246F} mutant, rescued cell migration ability in MCF-7/ADR cells. (B) The relative cell migration distance was quantified and plotted in the lower panel. Data are shown as mean ± SD; n = 6. Statistical analysis was performed by two-way ANOVA. ***P < 0.001 and ns P > 0.05 indicates no statistical significance.

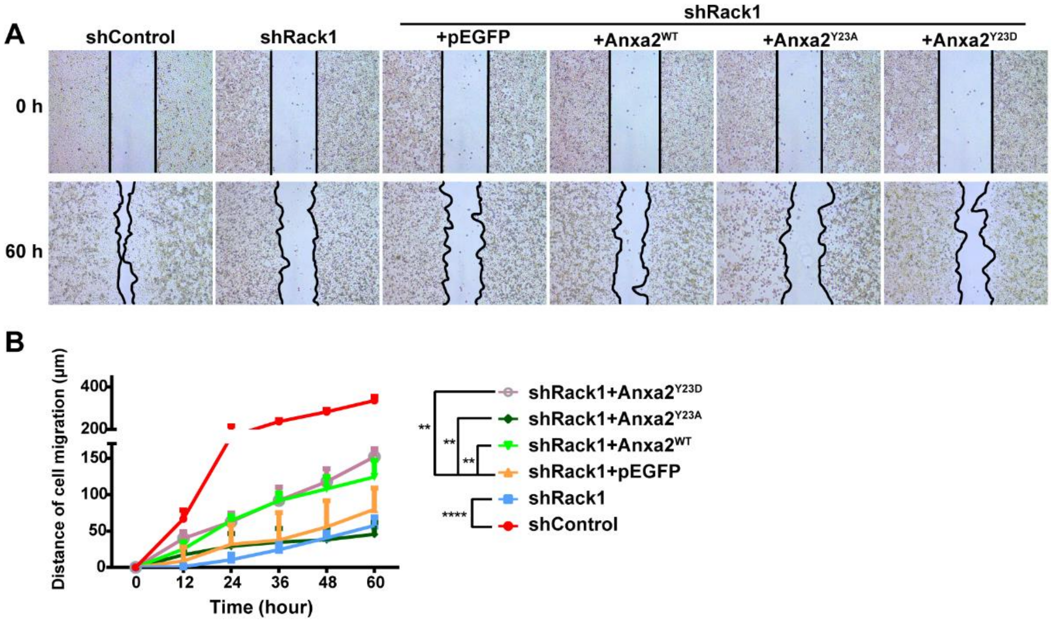

Figure S4. Increased expression of Anxa2^{WT} or Anxa2^{Y23D} in Rack1-silenced cells recovered cell migration ability. (A and B) Wound healing assay showed that the overexpression of Anxa2^{WT} or Anxa2^{Y23D}, not Anxa2^{Y23A}, partially rescued the cell migration ability in Rack1 silenced MCF-7/ADR cells. Data are shown as mean ± SD; n = 6. Statistical analysis was performed by two-way ANOVA. ****P < 0.0001 and **P < 0.01.

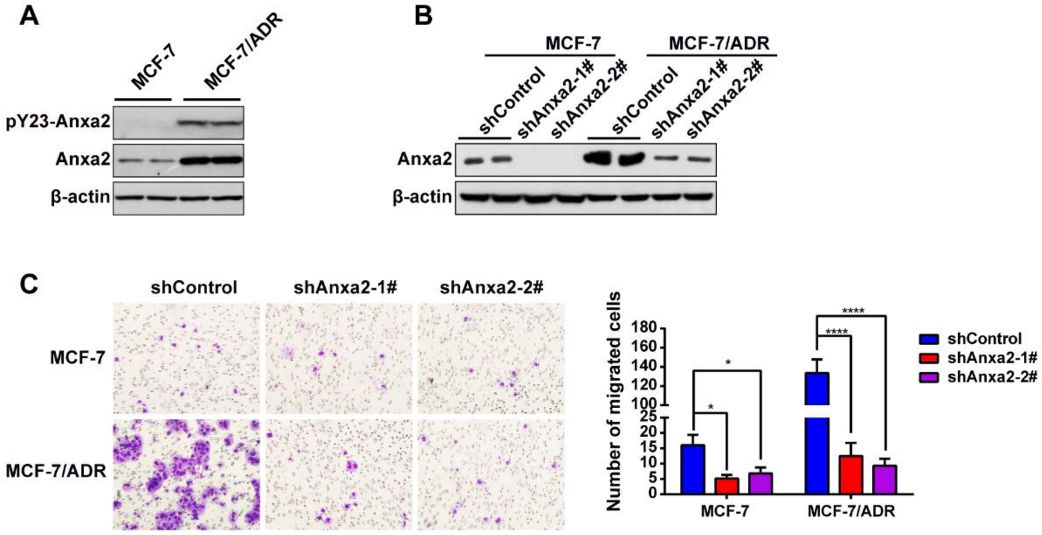

Figure S5. Silencing of Anxa2 expression attenuates migration ability in breast cancer cells. (A) Western blotting analysis showed that the expression of Anxa2 and pY23-Anxa2 in drug resistant MCF-7/ADR cells were elevated compared with that in MCF-7 cells. (B and C) Silencing the expression of Anxa2 in MCF-7 and MCF-7/ADR cells decreased migration ability as measured by transwell assay. Data were displayed as mean ± SD; n = 6. Statistical analysis was performed by one-way ANOVA. *P < 0.05, ****P < 0.0001 versus shControl.
